# Letter to the Editor

**DOI:** 10.3109/02813432.2013.846579

**Published:** 2013-12

**Authors:** Carson Adams, Daniel Cohen, Justin Schreiber, Robin Valpey, Kristy Benoit, Salomon Puyana, Xiaoran Zhang, Antoine Douaihy

**Affiliations:** University of Pittsburgh School of Medicine, Pittsburgh, Pennsylvania, United States. E-mail: adams.carson@medstudent.pitt.edu

The authors’ conclusion that their study [1] brings into question “the impact of motivational interviewing in terms of its ability to improve routine diabetes care in practice” is deserving of significant inquiry.

Our greatest concern is in regards to the quality of motivational interviewing (MI) delivered in the intervention group. Evidence has shown that, even though workshops can improve MI skills in trainees, those skills can be lost in as little as four months without continued feedback and coaching [2]. With this in mind, a significant limitation of this study is the lack of objective monitoring of the MI-based counseling sessions led by the nurses, calling into question the adherence of their counseling to the principles of MI. Additionally, this study combined the principles of MI with multiple other counseling techniques, including agenda setting, lifestyle counseling, and record keeping. These tools could have led to nurses conducting a more traditional counseling session where patients were provided information without the evocative and collaborative spirit of MI. The authors were not concerned about the quality of MI delivered in the study because “four training sessions were sufficient”; however, without objective analysis of counseling sessions and feedback (only 37% received feedback), it remains unclear whether skills developed in the four workshops were sustained throughout the study.

Without objective coding of counseling sessions to determine adherence to the principles of MI, it would be necessary to demonstrate the enduring skills of the nurses who participated in the workshops. In another paper [3] based on the same trial, the authors evaluated the strength of the nurses’ MI skills by comparing baseline interviews with one-year follow up interviews. Only two of 24 MI skills evaluated in the study showed statistical improvement one year after training. This calls into question the quality of the MI counseling provided by the nurses throughout the study given the evidence of “minimal improvement after MI training embedded in [their] comprehensive programme”. Yet, despite minimal improvement of skills, the study did demonstrate that when MI skills were implemented they were positively correlated with observed readiness for change, suggesting desirable results with successful use of MI skills.

Taking into account the lack of objective observation to ensure nurses’ fidelity to the principles of MI and the evidence of minimal improvement in skills one year after the nurses’ training, we believe that it is inaccurate to call into question the impact of MI in diabetes care.
